# Plasma amyloid and tau as dementia biomarkers in Down syndrome: Systematic review and meta‐analyses

**DOI:** 10.1002/dneu.22715

**Published:** 2019-09-11

**Authors:** Falah Alhajraf, Deborah Ness, Abdul Hye, Andre Strydom

**Affiliations:** ^1^ UCL Queen Square Institute of Neurology University College London London UK; ^2^ Al Amiri Hospital Kuwait City State of Kuwait; ^3^ Department of Forensic and Neurodevelopmental Sciences, Institute of Psychiatry, Psychology and Neuroscience King's College London London UK; ^4^ The LonDownS Consortium (London Down Syndrome Consortium) London UK; ^5^ The Maurice Wohl Clinical Neuroscience Institute, Institute of Psychiatry, Psychology and Neuroscience King's College London London UK

**Keywords:** Alzheimer's disease, biomarkers, dementia, Down syndrome, plasma/blood amyloid/Aβ

## Abstract

Individuals with Down syndrome (DS) are at high risk of developing Alzheimer's disease (AD). Discovering reliable biomarkers which could facilitate early AD diagnosis and be used to predict/monitor disease course would be extremely valuable. To examine if analytes in blood related to amyloid plaques may constitute such biomarkers, we conducted meta‐analyses of studies comparing plasma amyloid beta (Aβ) levels between DS individuals and controls, and between DS individuals with and without dementia. PubMed, Embase, and Google Scholar were searched for studies investigating the relationship between Aβ plasma concentrations and dementia in DS and 10 studies collectively comprising >1,600 adults, including >1,400 individuals with DS, were included. RevMan 5.3 was used to perform meta‐analyses. Meta‐analyses showed higher plasma Aβ_40_ (SMD = 1.79, 95% CI [1.14, 2.44], *Z* = 5.40, *p* < .00001) and plasma Aβ_42_ levels (SMD = 1.41, 95% CI [1.15, 1.68], *Z* = 10.46, *p* < .00001) in DS individuals than controls, and revealed that DS individuals with dementia had higher plasma Aβ_40_ levels (SMD = 0.23, 95% CI [0.05, 0.41], *Z* = 2.54, *p* = .01) and lower Aβ_42_/Aβ_40_ ratios (SMD = −0.33, 95% CI [−0.63, −0.03], *Z* = 2.15, *p* = .03) than DS individuals without dementia. Our results indicate that plasma Aβ_40_ levels may constitute a promising biomarker for predicting dementia status in individuals with DS. Further investigations using new ultra‐sensitive assays are required to obtain more reliable results and to investigate to what extent these results may be generalizable beyond the DS population.

## 1 INTRODUCTION

With a prevalence of approximately one in 1,000 live births in the United Kingdom (Morris & Springett, 2013), Down syndrome (DS) is the most common genetic cause of intellectual disability (Sherman, Allen, Bean, & Freeman, 2007).

Alongside the typical features of DS, several medical complications are associated with the condition, including dementia of Alzheimer's type. The clinical manifestation of dementia in DS resembles that occurring in Alzheimer's disease (AD) in the general population (Dekker et al., 2018; Startin et al., 2019), with slight differences in early presentation (Lautarescu, Holland, & Zaman, 2017). Although not all elderly individuals with DS receive a dementia diagnosis, nearly all individuals with full trisomy 21 aged 40 and older are found to have typical AD neuropathology (Davidson, Robinson, Prasher, & Mann, 2018), including extracellular amyloid plaques and intracellular neurofibrillary tangles, but also other features such as cerebral amyloid angiopathy (Mann et al., 2018). Compared to the general population, amyloid plaques usually occur earlier in DS individuals, and deposits of amyloid beta 1‐42 (Aβ_42_) in the cortex of DS subjects have even been discovered as early as at 12 years of age (Lemere et al., 1996). In addition, earlier studies using relatively insensitive assays have suggested that circulatory Aβ_42_ and amyloid beta 1‐40 (Aβ_40_) plasma levels are higher in DS individuals than in age‐matched controls, irrespective of their cognitive status (Mehta, Capone, Jewell, & Freedland, 2007; Mehta et al., 2003; Schupf et al., 2001; Tokuda et al., 1997).

In DS, the triplication of chromosome 21, where a critical gene encoding the amyloid precursor protein (APP) is located, leads to APP overexpression and thus increased accumulation of Aβ in the brains of affected individuals (Wiseman et al., 2015). Accumulation of Aβ plaques in the brain, which consist of Aβ peptides resulting from the cleavage of APP by β‐ and γ‐secretase enzymes (Chow, Mattson, Wong, & Gleichmann, 2010), plays an important role in AD pathogenesis. There are two major isoforms of Aβ peptides: the longer and less soluble Aβ_42_ which is more likely to aggregate into so‐called senile plaques and the shorter and more soluble Aβ_40_ (Jarrett, Berger, & Lansbury, 1993). The deposition of Aβ_42_ was found to precede the deposition of Aβ_40_ (Iwatsubo et al., 1994) and Aβ plaques can antedate the clinical manifestations of dementia in sporadic AD by a decade or more (Sperling et al., 2011).

The assumption that triplication of the APP gene causes AD pathology in DS is in line with rare case studies of individuals with partial trisomy of chromosome 21 who have only two copies of the APP gene, where post‐mortem neuropathological examinations revealed normal age‐related changes but no evidence of AD neuropathology (Doran et al., 2017; Prasher et al., 1998). However, the triplication of other genes on chromosome 21 aside from APP could also play a role in AD pathogenesis, as is suggested by findings of (a) differing amyloid deposition in animal model studies depending on the extent of the triplication (Wiseman et al., 2018), and (b) the apparent clinical and neuropathological differences between individuals with AD due to full trisomy 21 and those with the rare copy number variant resulting in APP duplication (Zis & Strydom, 2018).

The presence of intracellular neurofibrillary tangles consisting of hyperphosphorylated tau aggregates is another major neuropathological hallmark of AD (Grundke‐Iqbal et al., 1986). Abnormal hyperphosphorylation of tau proteins, which is mainly caused by the up‐regulation of protein kinases or the down‐regulation of protein phosphatases (Wang, Grundke‐Iqbal, & Iqbal, 2007), precipitates the disruption of tau function in stabilizing and maintaining the microtubules (Billingsley & Kincaid, 1997), resulting in their dismantling and the subsequent accumulation of tau aggregates in the form of straight or paired helical filaments known as neurofibrillary tangles (Alonso, Zaidi, Grundke‐Iqbal, & Iqbal, 1994; Alonso, Zaidi, Novak, Grundke‐Iqbal, & Iqbal, 2001). The density of the neurofibrillary tangles has been found to be directly associated with dementia severity (Farber et al., 2000; Tomlinson, Blessed, & Roth, 1970).

Although amyloid and tau proteins have both been extensively studied as potential biomarkers for AD, the findings remain inconclusive. Ideal AD biomarkers need to be minimally invasive and inexpensive to obtain, easy to use and analyse, rigorously validated, and they ought to possess high sensitivity and at least 85% specificity (Growdon et al., 1998). Despite the potential of identifying such biomarkers in cerebrospinal fluid (CSF), or using neuroimaging methods, such as Positron Emission Tomography (PET), and although these methods are both considered valid tools for aiding clinicians in diagnosing AD, the high cost of PET neuroimaging and the invasive nature of lumbar punctures for CSF analysis are serious disadvantages. Consequently, the need for reliable, less invasive, and inexpensive blood‐based biomarkers for AD in DS individuals is pivotal and could substantially improve the reliability of dementia diagnosis in the DS population, which usually is especially challenging due to pre‐existing impairments of intellectual abilities. Furthermore, the identification of biomarkers associated with dementia status and disease progression could facilitate clinical trials of new therapies with the potential to prevent or delay the onset of dementia or initial cognitive decline.

Therefore, this review summarizes results regarding (a) differences in Aβ and tau plasma levels between individuals with DS and controls, and (b) the relationship between these biomarkers and dementia status in DS individuals. In addition to providing an overview of the findings in this field, we conduct meta‐analyses to explore and estimate the potential of Aβ plasma levels as biomarkers for AD in DS.

## 2 METHODS

### 2.1 Search strategy

The literature search was performed using PubMed, Embase, and Google Scholar databases (see Tables S1 and S2 for our amyloid and tau search strategy, respectively). The following keywords were used: Down syndrome/trisomy 21, AD, dementia, plasma amyloid/tau, serum amyloid/tau, and blood amyloid/tau. The amyloid term was used interchangeably with Aβ, Aβ_1‐40_, Aβ_1‐42_, Aβ_40_, and Aβ_42_. The tau term was used interchangeably with plasma total tau, plasma phosphorylated tau, P‐T181, and phosphorylated tau at Serine 396 (P‐S396). The study period was restricted to the past 20 years and the results were filtered to display only full‐text, peer‐reviewed articles written in the English language. Only original studies were considered.

### 2.2 Study selection and data extraction

We included original studies which measured plasma Aβ_40_ and/or Aβ_42_ levels and/or the ratio of plasma Aβ_42_/Aβ_40_, as well as studies investigating either plasma total tau (t‐tau) levels or plasma levels of P‐S396 or at Threonine 181 (P‐T181) using techniques based on immunoassays. Studies had to compare biomarker plasma levels between adult (>16 years) DS individuals with and without dementia and/or between DS individuals and control subjects. Studies with a DS dementia group were only considered if (a) the difference between the mean age of demented and non‐demented participants did not exceed 20 years, and (b) an AD diagnosis was established by expert clinicians using one of the following set of criteria: The International Classification of Diseases (ICD)‐10 criteria, The Diagnostic and Statistical Manual of Mental Disorders, Fourth Edition (DSM‐IV) criteria, or The American Association of Mental retardation—International Association for Scientific Study of Intellectual Disability (AAMR‐IASSID) criteria.

**Figure 1:**
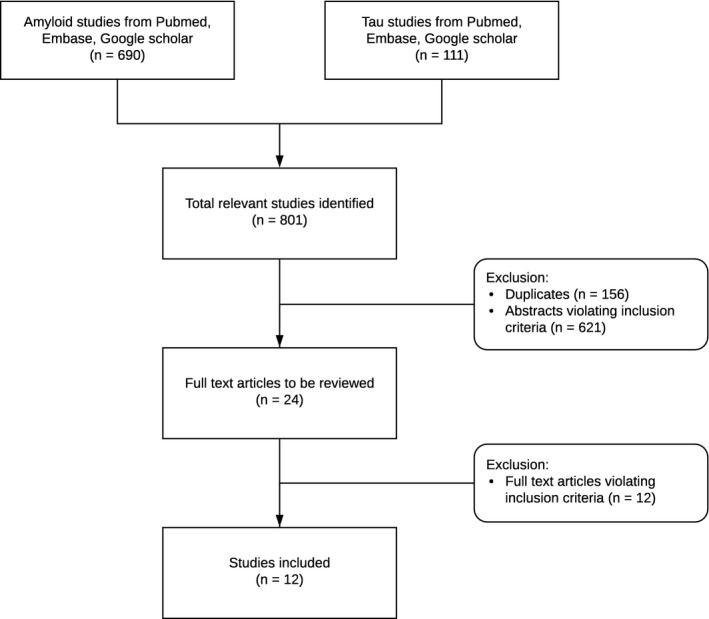
Flow chart diagram of study selection and inclusion

A total of 801 studies were identified by searching the electronic databases such as Pubmed, Google Scholar, and Embase (Figure 1). After duplicates had been removed, the abstracts of all the potentially suitable studies (*n* = 645) were carefully reviewed and studies were excluded if they did not report original data on amyloid or tau assays in samples of DS individuals. A total of 24 studies were determined to be eligible for full text review of which a final 12 fulfilled all inclusion criteria and were included in our review and meta‐analyses (Tables 1 and 2). Excluded studies and reasons for exclusion are listed in Table 3. The studies were conducted in multiple countries, including the United States, Italy, the UK, Spain, and the Netherlands. From each of the included original studies, the following data were extracted whenever available (all from one time point, also when longitudinal data were available as displayed in the last two columns of Table 1):

- Number of participants, including;
  o Number of males and females;
  o Number of demented and non‐demented DS individuals;
  o Number of healthy controls;
- Mean age of participants in each group ± standard deviation/standard error;
- Mean plasma Aβ_1‐42_ levels ± standard deviation/standard error;
- Mean plasma Aβ_1‐40_ levels ± standard deviation/standard error;
- Mean ratio of plasma Aβ_1‐42_/Aβ_1‐40_ ± standard deviation/standard error;
- Mean total tau plasma levels ± standard deviation/standard error;
- Methods applied to quantify plasma tau and amyloid levels.

**Table 1:** Overview of demographic data, plasma amyloid levels (pg/ml) and methodology of studies included in our meta‐analyses in alphabetic order of first author names

Study	Subjects	*m*	*f*	Age	Plasma Aβ_42_	Plasma Aβ_40_	Aβ_42_/Aβ_40_	Methods	Study design	Data used in meta‐analyses
	*n*	*n*	*n*	mean ± *SD*	mean ± *SD*	mean ± *SD*	mean ± *SD*			
Coppus et al. (2012)	DS 264	169	95	50.6 ± 4.4	51 ± 14.8	326.4 ± 101.3	0.16 ± 0.05	xMAP technology (Innogenetics)	Longitudinal clinical assessments: plasma samples collected at baseline only	Groups: DS versus DS.D DS.D = AD at baseline
	DS.D 62	36	26	54 ± 5.9	50 ± 17.5	352.3 ± 103.5	0.15 ± 0.06			Aβ values from baseline
Fortea et al. (2018)	DS 233				14.12 ± 3.18	343.83 ± 60.95		Simoa	Cross‐sectional design	Groups: DS versus DS.D DS = aDS +pDS DS.D = DS.D
	DS.D 49	28	21	54.88*, *	14.79 ± 3.42	384.39 ± 79.87				
Fortea et al. (2018)	DS 194	105	89	37.05*, *	14.22 ± 3.12	341.52 ± 60.19		Simoa	Cross‐sectional design	Groups: DS versus NC DS = aDS NC = NC
	NC 67	20	47	52.02*, *	9.41 ± 1.51	196.77 ± 26.58				
Head et al. (2011)	DS 26	17	9	45.1 ± 9.67	20.59 ± 8.27	275.76 ± 104.63	0.09 ± 0.102	ELISA: BAN50 + BC05/ BA27	Cross‐sectional design	Groups: DS versus DS.D
	DS.D 52	26	26	53.3 ± 5.05	23.78 ± 17.22	289.61 ± 98.96	0.08 ± 0.072			
Head et al. (2011)	DS 17	9	8	44.1 ± 5.77	32.88 ± 18.57	327.71 ± 117.80	0.10 ± 0.041	ELISA: BAN50 + BC05/ BA27	Cross‐sectional design	Groups: DS versus NC NC = Young Controls
	NC 11	5	6	46.5 ± 6.63	19.82 ± 6.72	196.97 ± 40.20	0.10 ± 0.033			
Iulita et al. (2016)	DS 21	10	11	34 ± 9.62	16.62 ± 7.79	228.4 ± 109.065		ELISA: Multi‐	Longitudinal	Groups: DS versus DS.D
	DS.D 10	6	4	52 ± 6.32	17.75 ± 7.27	258.5 ± 93.29		spot V‐PLEX Aβ peptide Panel (6E10)	design; plasma samples and clinical data collected at multiple visits	DS.D = AD at baseline Aβ values from baseline
	NC 31	16	15	38 ± 11.14	9.14 ± 5.01	90.17 ± 46.77				
Jones et al. (2009)	DS 39			48.8 ± 7.62	27.07 ± 11.43	121.32 ± 50.71		ELISA (commercial biosource)	Cross‐sectional design	Groups: DS versus DS.D
	DS.D 21			54 ± 5.045	27.85 ± 16.63	125.6 ± 84.14				
Matsuoka et al. (2009)	DS 145	89**	59**	54.2 ± 3.6**	1,527.03 ± 2,599.10	1,246.75 ± 1,662.34		ELISA: 82E1 + 1C3/ 1A10	Cross‐sectional design (using data from a longitudinal study on vitamin E)	Groups: DS versus DS.D
	DS.D 52	33	19	56 ± 3.9	1,887.34 ± 2,972.97	1,047.62 ± 1,389.61				
Prasher et al. (2010)	DS 83	52	31	49 ± 10.2	33.8 ± 15	177.8 ± 67.8	0.23 ± 0.23	ELISA: 6E10 + R165/ R162	Longitudinal cognitive assessments; plasma samples collected at last visit only	Groups: DS versus DS.D DS.D = AD at follow‐up
	DS.D 44	30	14	56.8 ± 4.9	33.2 ± 15.9	179.6 ± 59.7	0.21 ± 0.13			
Schupf et al. (2001)	DS 97			51.9 ± 6.6	22.4 ± 6.1	132.1 ± 44.4		ELISA: 6E10 + R165/ R162	Cross‐sectional design	Groups: DS versus DS.D
	NC 64			51.5 ± 7.1	14.2 ± 4.5	84.7 ± 19.6				
Schupf et al. ( 2010)	DS 164	55	109	50.3 ± 5.2	33.4 ± 8.59	150.1 ± 53.79	0.25 ± 0.13	ELISA: 6E10 + R165/ R162	Longitudinal design with DS subjects without AD at baseline; plasma samples collected at multiple visits	Groups: DS versus DS.D DS.D = Incident AD at follow‐up
	DS.D 61	18	43	53.7 ± 5.4	25.8 ± 21.77	172.1 ± 52.33	0.16 ± 0.08			Aβ values from follow‐up
Startin et al. (2019)	DS 24	17	7	45.25 ± 10.90	25.42 ± 8.46	308.93 ± 105.75	0.086 ± 0.019	Simoa	Cross‐sectional design	Groups: DS versus DS.D
	DS.D 7	5	2	52 ± 10.36	27.07 ± 8.19	363.71 ± 116.14	0.076 ± 0.015			
Startin et al. (2019)	DS 31	22	9	46.77 ± 10.99	25.79 ± 8.29	321.30 ± 108.69	0.083 ± 0.019	Simoa	Cross‐sectional design	Groups: DS versus NC
	NC 27	16	11	49.26 ± 10.4	15.72 ± 7.43	148.39 ± 75.75	0.110 ± 0.023			

Note

The studies by Head et al. (2011), Fortea et al. (2018), and Startin et al. (2019) were split into two rows each due to the use of different samples for comparisons of DS versus NC and DS.D versus DS groups. All amyloid values have been converted to pg/ml whenever necessary, using 1 pg/mL = 0.222 pmol/L for Aβ_42_ and 0.231 pmol/L for Aβ_40._ Values reported as standard errors (SE) were converted to *SD*: *SD* = SE * √*n*.

Abbreviations: AD = Alzheimer's disease; aDS = DS subjects who are asymptomatic for AD; Down syndrome; DS.D = Down syndrome with dementia; NC = normal controls, pDS = DS subjects who are in the prodromal stage of AD but do not fulfil criteria for AD diagnosis; *SD* = standard deviation.

* Indicates median values;

** Values were calculated for a total of 148 subjects, not 145 (3 were subsequently excluded).

**Table 2:** Overview of demographic data, P‐T181 levels (pg/ml), and methodology of studies included in our review in alphabetic order of first author names

Study	Subjects (*N*)	Male (*N*)	Female (*N*)	Age (Mean ± *SD*)	Plasma t‐tau (Mean ± *SD*)	Plasma P‐T181 (Mean ± *SD*)	Methods
Kasai et al. (2017)	DS 21	11	10	33.1 ± 11.9	0.643 ± 0.493		Simoa
	NC 22	12	10	37.4 ± 12	0.470 ± 0.232		
Tatebe et al. (2017)	DS 20	10	10	34.0 ± 11.5		0.767 ± 1.26	Simoa
	NC 22	12	10	37.4 ± 12		0.042 ± 0.071	
Fortea et al. (2018)	DS 233				3.27 ± 4.77		Simoa
	DS.D 49	28	21	54.88*, *	3.85 ± 1.50		
Fortea et al. (2018)	NC. 67	20	47	52.05*, *	3.95 ± 5.07		Simoa
	DS 194	105	89	37.05*, *	3.3 ± 5.2		
Startin et al. (2019)	DS 31	22	9	46.77 ± 10.99	2.03484 ± 2.508707		Simoa
	NC 27	16	11	49.26 ± 10.4	2.38037 ± 2.525724		
Startin et al. (2019)	DS 24	17	7	45.25 ± 10.90	1.82500 ± 2.443661		Simoa
	DS.D 7	5	2	52.00 ± 10.36	2.75429 ± 2.792382		

Note

The studies by Fortea et al. (2018), and Startin et al. (2019) were split into two rows each due to the use of different samples for comparisons of DS versus NC and DS.D versus DS groups. Values reported as standard errors (SE) were converted to *SD*: *SD* = SE * √*n*.

Abbreviations: DS = Down syndrome; DS.D = Down syndrome with dementia; NC = normal controls, *SD* = standard deviation.

* Indicates median values.

**Table 3:** Excluded studies and reasons for exclusion

Study	Reason of exclusion
1. Obeid, Hübner, Bodis, and Geisel (2016)	Age of subjects <16 years
2. Rafii et al. (2017)	Pilot study with no clear numerical results of mean plasma amyloid levels ± *SD* presented
3. Matsubara et al. (2004)	Measured levels of the soluble form of amyloid; no clear numerical results of mean plasma amyloid levels ± *SD* presented
4. Cavani et al. (2000)	Measured levels of the soluble form of amyloid
5. Mehta et al. (2001)	No clear numerical results of mean plasma tau levels ± *SD* presented
6. Mehta et al. (2007)	Age of subjects <16 years
7. Tokuda et al. (1997)	Study older than 20 years (published before 1998)
8. Lee, Chien, and Hwu (2017)	Dementia diagnosis on the basis of a screening tool (Adaptive Behavior Dementia Questionnaire [ABDQ])
9. Hamlett et al. (2017)	Measured neuronal exosome contents, not plasma concentrations
10. Mehta et al. (1998)	Reported only median values, not means
11. Mehta et al. (2003)	Reported only median values, not means
12. Schupf et al. (2007)	Sample overlap with Schupf et al. (2010)

### 2.3 Participants

A total of 1,682 adult participants above the age of 16 were included in our meta‐analyses, of whom 200 were normal controls and 1,482 were individuals with DS. Of the DS individuals, 369 had a diagnosis of dementia and 1,113 did not. Additional information on age and sex of included participants per study is listed in Tables 1 and 2.

### 2.4 Statistical analysis

All meta‐analyses were conducted using Review Manager (RevMan) version 5.3 (The Cochrane Collaboration, 2014). Using a random effects model, calculations were performed with standardized mean differences (SMD) in order to obtain effect sizes and 95% confidence intervals (CI) for studies comparing DS individuals versus healthy controls, and demented versus non‐demented DS individuals regarding plasma levels of the following peptides: Aβ_42_, Aβ_40_, and Aβ_42_/Aβ_40_ ratio. The statistical heterogeneity between studies was measured using a *I* ^2^ tests. Funnel plots were created to be able to detect publication bias among the studies (Figures [Link], [Link], [Link], [Link], [Link]). The number of studies reporting plasma tau levels was too limited to perform meta‐analyses.

## 3 RESULTS

### 3.1 Amyloid

Studies which compared DS individuals and healthy controls consistently report higher plasma Aβ_42_ and Aβ_40_ levels DS individuals (Table 1). Differences between DS individuals and controls regarding the ratio of plasma Aβ_42_/Aβ_40_ were investigated in two studies (Table 1) and while Head et al. (2011) failed to detect a significant difference between the groups, Startin et al. (2019) found higher Aβ_42_/Aβ_40_ ratios in controls.

Regarding differences in plasma Aβ peptide levels according to dementia status among DS individuals, results were more mixed (Table 1): the study by Matsuoka et al. (2009) suggested that instead of the respective plasma levels, it was the heightened plasma Aβ_42_/Aβ_40_ ratio which was associated with dementia status in DS. Interestingly, the study also reported a correlation between plasma Aβ_42_ levels and severity of intellectual disability, suggesting a potential conflation between dementia diagnosis and degree of intellectual disability. Prasher, Sajith, Mehta, Zigman, and Schupf (2010) reported that dementia duration of over 4 years was associated with higher Aβ_42_/Aβ_40_ ratios, and with decreased plasma Aβ_40_ levels. However, when directly comparing demented and non‐demented DS participants, no difference in plasma amyloid levels was evident. This is in alignment with the findings from Head et al. (2011), Iulita et al. (2016), and Jones, Hanney, Francis, and Ballard (2009), which revealed no significant differences in plasma amyloid levels between demented and non‐demented individuals with DS. The lack of significant findings in these studies may be partly due to the applied assays and/or the used antibodies (Table 1), and the limited power due to small to moderate sample sizes. It is important to bear in mind that the application of modern ultra‐sensitive technology or the incorporation of bigger samples may have yielded different results. 

Schupf and colleagues (2001, 2007, 2010) have conducted three studies which focused on the relationship between amyloid plasma levels and dementia in DS. Despite using the same amyloid quantification methodology in each of the studies and potentially overlapping participants, the results were variable. Two of these studies indicated that demented DS individuals had significantly higher plasma levels of Aβ_42_, but not Aβ_40_ (Schupf et al., 2007, 2001). Contrarily, in the third study (Schupf et al., 2010), they found that DS individuals who developed dementia over the course of the study had higher plasma Aβ_40_ but lower Aβ_42_ levels as well as lower Aβ_42_/Aβ_40_ ratios. A different and more sensitive approach was embraced by Coppus and colleagues (2012), who were the first to utilize the Multi‐Analyte Profiling (xMAP) technology to measure plasma Aβ levels in people with DS. Contrary to the findings reported above, this study indicated that DS individuals with dementia had significantly higher plasma Aβ_40_ levels than those without dementia, and that elevated plasma levels of both Aβ_40_ and Aβ_42_ were associated with an increased risk of developing dementia. Interestingly, no significant difference was found between the two groups in terms of plasma Aβ_42_ levels and Aβ_42_/Aβ_40_ ratios in this study (Coppus et al., 2012).

More recently, several studies used the Single Molecule Array (Simoa)  technology to measure plasma amyloid and tau levels in DS individuals (Tables 1 and 2). This ultra‐sensitive new technology can reliably detect important disease‐related proteins with substantially higher sensitivity, and studies which used SIMOA to quantify t‐tau or Neurofilament light (NfL) in AD have highlighted its feasibility and advantages (Fortea et al., 2018; Mattsson, Andreasson, Zetterberg, & Blennow, 2017; Weston et al., 2017). One of these recent studies which was conducted by Fortea et al. (2018), reported that plasma Aβ_40_ levels were significantly higher in demented DS individuals compared to non‐demented DS individuals. However, no significant association between plasma Aβ_42_ levels and dementia status in DS was detected. Startin and colleagues (2019), who also used ultra‐sensitive methods, reported both significantly increased plasma Aβ_40_ and Aβ_42_ levels as well as lower Aβ_42_/Aβ_40_ ratios in DS individuals compared to controls and compared to individuals with sporadic AD, however, due to the small number of DS individuals with dementia in this study, no direct comparison between demented and non‐demented DS individuals was calculated.

### 3.2 Meta‐analysis I: Aβ levels in individuals with DS versus healthy controls

**Figure 2:**
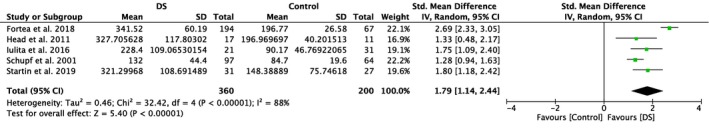
Meta‐analysis of studies comparing plasma Aβ_40_ levels of individuals with DS and healthy controls. Abbreviations: DS = Down syndrome, *SD* = standard deviation, CI = Confidence Interval, Std. = Standardized [Color figure can be viewed at http://www.wileyonlinelibrary.com]

**Figure 3:**
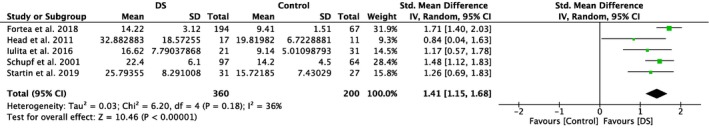
Meta‐analysis of studies comparing plasma Aβ_42_ levels of individuals with DS and healthy controls. Abbreviations: DS = Down syndrome, *SD* = standard deviation, CI = Confidence Interval, Std. = Standardized [Color figure can be viewed at http://www.wileyonlinelibrary.com]

Five studies included in this meta‐analysis compared plasma amyloid levels of DS individuals with those of healthy controls (Table 1). Meta‐analyses showed significantly higher plasma Aβ_40_ levels in DS individuals compared to healthy controls (SMD = 1.79, 95% CI [1.14, 2.44], *Z* = 5.40, *p* < .00001; Figure 2). A significant difference with higher levels in the DS group was also detected for plasma amyloid Aβ_42_ (SMD = 1.41, 95% CI [1.15, 1.68], *Z* = 10.46, *p* < .00001), which is illustrated in Figure 3. Notably, results were highly and moderately heterogeneous, with *I* ^2^ scores of 88% and 36%, respectively.

### 3.3 Meta‐analysis II: Aβ levels in demented versus non‐demented individuals with DS

**Figure 4:**
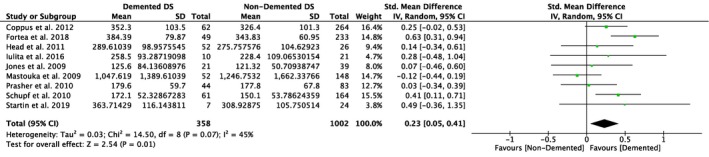
Meta‐analysis of studies comparing plasma Aβ_40_ levels of DS individuals with and without dementia. Abbreviations: DS = Down syndrome, *SD* = standard deviation, CI = Confidence Interval, Std. = Standardized [Color figure can be viewed at http://www.wileyonlinelibrary.com]

**Figure 5:**
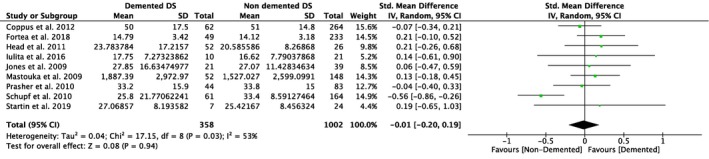
Meta‐analysis of studies comparing plasma Aβ_42_ levels of DS individuals with and without dementia. Abbreviations: DS = Down syndrome, *SD* = standard deviation, CI = Confidence Interval, Std. = Standardized [Color figure can be viewed at http://www.wileyonlinelibrary.com]

**Figure 6:**
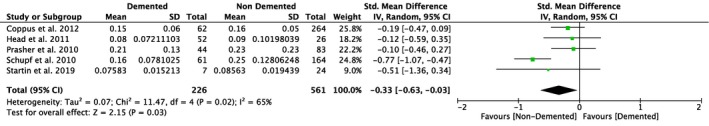
Meta‐analysis of studies comparing plasma Aβ_42_/Aβ_40_ ratios of DS individuals with and without dementia. Abbreviations: DS = Down syndrome, *SD* = standard deviation, CI = Confidence Interval, Std. = Standardized [Color figure can be viewed at http://www.wileyonlinelibrary.com]

Nine studies compared plasma Aβ_40_ as well as Aβ_42_ levels in DS individuals with and without dementia, and five of them also investigated differences in the ratio of Aβ_42_/Aβ_40_ (Table 1). Our meta‐analyses revealed significant differences between non‐demented and demented individuals with DS in plasma Aβ_40_ levels (SMD = 0.23, 95% CI [0.05, 0.41], *Z* = 2.54, *p* = .01; Figure 4), but not in plasma Aβ_42_ levels (SMD = −0.01, 95% CI [−0.20, 0.19], *Z* = 0.08, *p *= .94; Figure 5). Moreover, we found a significant association between Aβ_42_/Aβ_40_ ratios and dementia status with lower ratios in DS individuals with dementia compared to DS individuals without dementia (SMD = −0.33, 95% CI [−0.63, −0.03], *Z* = 2.15, *p* = .03; Figure 6). All these results were moderately heterogeneous with *I* ^2^ scores of 45%, 53%, and 65%, respectively.

### 3.4 Confounders

#### 3.4.1 Demographic variables

Demented DS individuals included in these studies tended to be older on average than non‐demented individuals with DS (Table 1). However, of the studies which evaluated the relationship between age and plasma Aβ levels, none was able to detect a significant association between these two variables. Furthermore, none of the studies that investigated this detected any significant differences in Aβ peptide levels between males and females. Moreover, articles which investigated whether plasma Aβ levels differ based on severity of intellectual disability did not detect any significant relationship.

#### 3.4.2 Apo E allele

The *APOE ε4* allele is considered a significant risk factor for dementia and several studies examined the effect of *APOE* alleles on plasma Aβ levels, with inconsistent and variable results. Most found no association between *APOE* alleles and plasma amyloid levels. However, Coppus et al. (2012) found *APOE ε4* allele status to be associated with higher plasma Aβ_42_ levels. The study by Head et al. (2011) suggested that elevated levels of plasma Aβ_40_ rather than Aβ_42_ are associated with *APOE ε4*.

#### 3.4.3 Duration of dementia and comorbidities

Prasher and colleagues (2010) reported that increased duration of dementia was associated with elevated levels of plasma Aβ_42,_ a higher Aβ_42_/Aβ_40_ ratio, as well as decreased plasma Aβ_40_ levels. Conversely, no relationship was found between plasma Aβ concentrations and the duration of dementia in the study conducted by Jones et al. (2009).

### 3.5 Predictive validity of baseline biomarkers for longitudinal cognitive decline or onset of dementia

There were too few longitudinal studies to allow for meta‐analysis of changes in biomarker levels. Only two of the included studies (Iulita et al., 2016; Schupf et al., 2010) collected more than one plasma sample. Iulita and colleagues (2016) reported no consistent significant change in plasma Aβ levels over 12 months on a group level (DS subjects and DS subjects with dementia), however their findings suggest an association between a decrease in plasma Aβ_1‐42_ and Aβ_1‐40_ levels in asymptomatic DS subjects over 2 years and more pronounced cognitive decline, while increased or stable Aβ levels over the same time period were not associated with cognitive outcome. On the other hand, the study by Schupf et al. (2010) found declining levels of plasma Aβ_1‐42_ levels, a declining plasma Aβ_1‐42_/Aβ_1‐40_ ratio, and increasing Aβ_1‐40_ levels to be related to conversion to AD.

Another study attempted to relate longitudinal cognitive decline to baseline biomarker levels (Coppus et al., 2012). They report that individuals with the highest Aβ_1‐40_ and AB_1‐42_ levels had higher risk of developing dementia over time. Moreover, Iulita et al. (2016) found higher plasma Aβ_1‐40_ and AB_1‐42_ levels at baseline to be associated with a higher rate of cognitive decline in at follow‐up in non‐demented DS individuals. Schupf and colleagues (2010) did not investigate plasma Aβ levels at baseline in relation to change in cognitive performance over time.

### 3.6 Tau

#### 3.6.1 Comparison between DS individuals and normal controls

Studies comparing plasma tau levels in people with DS and healthy controls are summarized in Table 2. Three studies examined total plasma tau levels and one focused on the phosphorylated form of plasma tau (P‐T181). All studies used Simoa^®^ for tau quantification.

Fortea et al. (2018) found plasma t‐tau levels to be significantly elevated in DS individuals compared to controls. In line with these results are the findings by Tatebe et al. (2017), who report that individuals with DS had significantly higher levels of P‐T181 compared to healthy controls. Similarly, the study by Kasai et al. (2017), which used the same sample as Tatebe et al. (2017), detected higher levels of plasma t‐tau in individuals with DS than in healthy controls. In addition, both studies found a significant positive correlation between age and plasma tau levels. Finally, although Startin et al. (2019) report plasma t‐tau levels, they did not calculate any group comparisons for this biomarker.

#### 3.6.2 Comparison between demented and non‐demented DS individuals

Only two studies included in this review look at plasma tau levels and dementia status in DS individuals: Fortea et al. (2018) reported that demented DS individuals have higher levels of plasma t‐tau relative to non‐demented DS individuals. Startin et al. (2019) do not report any group comparisons for this biomarker.

## 4 DISCUSSION

To the best of our knowledge, this is the first meta‐analysis to specifically focus on plasma amyloid and tau levels and their association with dementia in individuals with DS. It encompasses a total of 1,482 subjects with DS, as well as 200 normal healthy controls.

Overall, individuals with DS were found to have higher plasma Aβ_40_ and Aβ_42_ levels than healthy controls. Moreover, our meta‐analyses revealed statistically significant differences between DS individuals with and without dementia: Individuals with DS who had a dementia diagnosis were found to have higher plasma Aβ_40_ levels and lower Aβ_42_/Aβ_40_ ratios than non‐demented DS individuals. However, no significant association between plasma Aβ_42_ levels and dementia status was found. Studies' heterogeneity was moderate to high likely due to differences in assays used. On the contrary, all the studies which investigated plasma tau levels used ultrasensitive methods (Simoa), but due to the small number of them it was not possible to conduct any meta‐analyses.

The increased levels of plasma Aβ_40_ and Aβ_42_ in people with DS are most likely a consequence of the overexpression of the *APP* gene due to the triplication of chromosome 21. Since *APP* is a dosage‐dependent gene, amyloid plasma levels are expected to increase 1.5‐fold in the presence of a third copy of the gene (Amano et al., 2004; Lyle, Gehrig, Neergaard‐Henrichsen, Deutsch, & Antonarakis, 2004; Sultan et al., 2007). While plasma Aβ_42_ levels were in line with this prediction, plasma Aβ_40_ levels in the studies included here were overall slightly higher than expected and suggested an almost 1.8‐fold increase in DS individuals compared to healthy controls. One potential explanation of this finding might be the independent role of other triplicated genes on chromosome 21 aside from *APP*, which may influence APP processing and amyloid clearance (Wiseman et al., 2018), with a potential shift in Aβ subtypes (Buss et al., 2016; Zis & Strydom, 2018).

Different methods and variable techniques were implemented to measure plasma Aβ and tau levels and may in turn have influenced the results of the individual studies included in this review. Although all the studies used ELISA assays, it is important to acknowledge that a major issue with these assays is the lack of sensitivity to detect minimal amounts of plasma Aβ and tau peptides. While these assays have been validated and used extensively in CSF AD biomarker studies, Aβ levels in blood plasma are substantially lower than Aβ levels in CSF. Therefore, studies incorporating new ultra‐sensitive technologies, including IMR, Simoa, xMAP technology and IP‐MS (Lue, Guerra, & Walker, 2017), which can improve the accuracy of the results, are extremely valuable. These ultra‐sensitive methods were used by three Aβ studies included in our systematic review: Coppus et al. (2012) used xMAP technology, while Fortea et al. (2018) and Startin et al. (2019) used Simoa.

In addition to methodological differences, other factors may contribute to discrepancies between study results on plasma amyloid and tau levels, including age, *APOE* allele status, duration of dementia, and other genetic risk factors. While the included studies showed no association between age and plasma amyloid levels, results by a study by Schupf et al. (2007) showed that plasma Aβ_42_ increased with age in a DS population. In sporadic AD, in contrast, age was found to be more consistently associated with increased levels of plasma Aβ peptides (Fukumoto et al., 2003; Gabelle et al., 2015; Hanon et al., 2018; Li et al., 2015), and only a few articles reported no significant correlation (Lövheim et al., 2017; Mehta, Pirttilab, Patricka, Barshatzkya, & Mehta, 2001). These discrepancies may be due to a non‐linear relationship between age and Aβ levels in individuals with AD, with levels increasing prior to dementia diagnosis, but decreasing again during later stages of the disease. The association between Aβ levels and duration of dementia in DS was only investigated in two studies included in this systematic review: while Jones and colleagues (2009) found no association between dementia duration and plasma Aβ levels, the study by Prasher et al. (2010) revealed that longer dementia duration was associated with both increased plasma Aβ_42_ levels and Aβ_42_/Aβ_40_ ratios, and with decreased plasma Aβ_40_ levels. Although we could not control for dementia duration in our meta‐analyses, these results are contradictory to the findings of our meta‐analysis of higher levels of plasma Aβ_40_ and lower Aβ_42_/Aβ_40_ ratios in DS individuals with dementia compared to those with no dementia. Nevertheless, the studies included here recruited DS individuals at all stages of dementia which can partially explain apparent differences in findings and could hence also have obscured the findings of the current meta‐analysis. To be able to address this issue in future meta‐analysis, more longitudinal studies are required for clarification regarding the association between changes in plasma biomarker levels over time and onset, duration as well as severity of dementia.

The *APOE ε4* allele is a strong genetic risk factor for AD and *APOE ε4* allele carriers were found to have more Aβ accumulations in the brain compared to *APOE ε4* non‐carriers (Kok et al., 2009; Schmechel et al., 1993). Certainly, this prompts questions about whether *APOE* carrier status affects plasma Aβ levels in the DS population investigated here, but results are conflicting as some of the included studies did not report any association between *APOE* alleles and amyloid plasma levels.

Consistency of results has been noted to be an issue in AD biomarker studies, and sample handling, processing, and other laboratory factors can substantially contribute to discrepancies in study results (O’Bryant et al., 2017). An illustrative example is the research by Schupf and colleagues who conducted three studies in DS individuals in 2001, 2007, and in 2010, and applied the same methods and antibody assays to quantify plasma Aβ levels in each study. Nevertheless, the results were inconsistent and conflicting. This demonstrates the complexity of properly standardizing methods even in the same institution, and it highlights potential effects of sample heterogeneity, and of power limitations.

It is also important to consider that plasma amyloid peptides are not only of central nervous system (CNS) origin but have also been found to be produced by platelets, vascular walls, and skeletal muscles (Askanas, Engel, & Nogalska, 2015; Kuo et al., 2000; Li et al., 1998; Nostrand, 2016). Particularly platelets are regarded as an important source of blood amyloid peptides (Chen, Inestrosa, Ross, & Fernandez, 1995; Kucheryavykh et al., 2017). This can influence plasma Aβ levels and obscure the relationship between plasma Aβ levels and Aβ brain pathology. Fortunately, this is less of a problem when investigating plasma tau levels as tau is more CNS‐specific, and clearly more research is needed regarding tau as a biomarker for AD in DS. However, tau is not specific to AD pathology, and blood tau levels have been shown to be increased in other CNS pathologies, such as traumatic brain injury and cerebral infarction (Bielewicz, Kurzepa, Czekajska‐Chehab, Stelmasiak, & Bartosik‐Psujek, 2010; Liliang et al., 2010).

The accuracy of the clinical diagnosis of dementia could be another important contributor leading to heterogeneous results in studies on AD biomarkers in DS. To minimize this effect, our inclusion criteria required that the diagnosis of dementia had been made by an expert clinician using ICD‐10, DSM‐IV, or AAMR‐IASSID criteria. Although dementia diagnosis has been shown to be reliable in DS individuals (Sheehan et al., 2015), diagnosis may vary between health institutions and between clinicians.

### 4.1 Strengths and limitations

To the best of our knowledge, our study provides the first meta‐analysis of studies investigating plasma Aβ levels in DS individuals with and without dementia and compared to controls. Strict inclusion criteria were applied to ensure comparability of all included articles. Most included studies had rather small sample sizes limiting their statistical power, hence the need for a meta‐analysis.

Nevertheless, there are limitations to our study. First and foremost, different methods were used to quantify plasma Aβ levels in different studies; however, these were all based on immunoassay‐based technologies and inclusion of studies was thus justified. Furthermore, we incorporated the use of SMD in our meta‐analyses to account for differences in measurements. However, our analyses were limited by the rather small number of available studies. Finally, the data used in the meta‐analyses was cross‐sectional and detecting age‐ or AD‐specific subtle changes within subjects was not possible. Longitudinal study designs are particularly valuable when investigating changes associated with pathology over time, hence having more studies of this type could substantially further our understanding of the link between Aβ and tau plasma levels and the development of cognitive decline and AD in individuals with DS.

### 4.2 Future directions

Identifying reliable biomarkers which reflect cognitive decline and/or dementia status in individuals with DS is a crucial step in improving the diagnosis and management of AD and other forms of dementia in affected individuals. Not only could such a biomarker facilitate measuring and monitoring the pathological changes associated with dementia, but it may also help assess the effectiveness of new therapies in clinical trials. This knowledge could likely be extended beyond DS to other populations at high risk for AD and may ultimately help identify patients in the preclinical phase when brain cells can still be protected. This phase is thought to be the window of opportunity for intervention because at this stage neuronal death and the manifestation of the disease can theoretically still be prevented.

Although this review has highlighted differences in Aβ levels regarding dementia status in DS individuals, further studies are required to reliably use plasma Aβ or tau as biomarkers for dementia in DS. Especially longitudinal studies investigating the association between plasma amyloid and tau levels and the development of clinical dementia need to be conducted, and the relationships between plasma Aβ and tau levels and AD pathology in DS individuals' brains should additionally be explored using neuroimaging studies. In order to be able to reliably use plasma Aβ and tau levels as biomarkers for dementia in DS and to predict disease progression, it will have to be conclusively shown that they do not only reflect AD neuropathology, but also clinical progression over time. A combination of plasma biomarkers, including NfL, which has recently been shown to be related to dementia status and age in DS individuals (Fortea et al., 2018; Strydom et al., 2018), markers of oxidative stress (Coppus, Fekkes, Verhoeven, Tuinier, & van Duijn, 2010; Zis, Dickinson, Shende, Walker, & Strydom, 2012; Zis et al., 2014), and of inflammation (Startin et al., 2019) could be explored for improved prediction. Moreover, it has recently been revealed that exploring the role of smaller amyloid fragments in plasma using high‐performance immunoprecipitation combined with mass spectrometry (IP‐MS) may also be a promising approach for future research (Nakamura et al., 2018). The study showed significant association between plasma amyloid and both CSF biomarkers and brain amyloid using PIB‐PET with up to 90% accuracy. Similar results were observed by (Ovod et al., 2017) used a liquid chromatography MS (LC‐MS) approach to quantify amyloid. These studies investigated the correlation between plasma amyloid and amyloid deposition and would be beneficial to add to the clinical diagnosis of dementia as an important parameter in future research in the DS population.

### 4.3 Conclusion and recommendations

The risk of dementia is severely elevated in the DS population. Early diagnosis of dementia is crucial for early intervention and better disease management. Plasma tau and Aβ levels have the potential to serve as dementia biomarkers in individuals with DS. Higher baseline levels of plasma Aβ_40_ and Aβ_42_ were found in individuals with DS relative to healthy controls. Moreover, our meta‐analyses indicate associations between plasma Aβ_40_ levels as well as Aβ_42_/A_β40_ ratios and dementia status in DS individuals.

Finally, we identified variability in the results across the currently existing literature on biomarkers, which clearly highlights the need for more and larger, ideally longitudinal studies investigating the relationship between dementia and plasma Aβ and tau levels in DS. We also recommend the use of new ultrasensitive amyloid and tau quantification methods in order to yield more accurate and ultimately more reliable results, increasing the comparability of studies. Notably, it is of utter importance to standardize laboratory settings and processes of measuring plasma Aβ and tau levels to reduce the variability of results and to ensure their validity and reproducibility.

## CONFLICT OF INTEREST

All authors declare no conflicts of interest.

5

## Data Availability

Data sharing is not applicable to this article as no new data were created or analyzed in this study.
