# Supplementary material for: Plasma amyloid and tau as dementia biomarkers in Down syndrome: Systematic review and meta‐analyses
Source: Dev Neurobiol. 2019 Sep 11;79(7):684–98. doi: 10.1002/dneu.22715 (PMC6790908; doi:10.1002/dneu.22715)
Supplement: Supplementary file 6 [file DNEU-79-684-s006.docx]

# Supplemental Material

| Search | Query | Items found |
| --- | --- | --- |
| #8 | Search **(((("Down Syndrome"[Mesh]) OR Down* Syndrome)) AND (("Alzheimer Disease"[Mesh]) OR (Alzheimer* Disease OR dementia))) AND (plasma amyloid OR serum amyloid OR blood amyloid OR plasma Aβ OR Aβ 1-42 or Aβ 1-40 OR Aβ 42 OR Aβ 40)** Sort by: **Best Match** | [284](https://www.ncbi.nlm.nih.gov/pubmed/?cmd=HistorySearch&querykey=24) |
| #7 | Search **plasma amyloid OR serum amyloid OR blood amyloid OR plasma Aβ OR Aβ 1-42 or Aβ 1-40 OR Aβ 42 OR Aβ 40** Sort by: **Best Match** | [30305](https://www.ncbi.nlm.nih.gov/pubmed/?cmd=HistorySearch&querykey=23) |
| #6 | Search **("Alzheimer Disease"[Mesh]) OR (Alzheimer* Disease OR dementia)** Sort by: **Best Match** | [227405](https://www.ncbi.nlm.nih.gov/pubmed/?cmd=HistorySearch&querykey=22) |
| #5 | Search **Alzheimer* Disease OR dementia** Sort by: **Best Match** | [227405](https://www.ncbi.nlm.nih.gov/pubmed/?cmd=HistorySearch&querykey=21) |
| #4 | Search **"Alzheimer Disease"[Mesh]** Sort by: **Best Match** | 84328 |
| #3 | Search **("Down Syndrome"[Mesh]) OR Down* Syndrome** Sort by: **Best Match** | [44070](https://www.ncbi.nlm.nih.gov/pubmed/?cmd=HistorySearch&querykey=19) |
| #2 | Search **Down* Syndrome** Sort by: **Best Match** | [44070](https://www.ncbi.nlm.nih.gov/pubmed/?cmd=HistorySearch&querykey=18) |
| #1 | Search **"Down Syndrome"[Mesh]** Sort by: **Best Match** | [23226](https://www.ncbi.nlm.nih.gov/pubmed/?cmd=HistorySearch&querykey=17) |

**Supplemental Table 1.** Search strategy for amyloid studies in Pubmed

| Search | Query | Items found |
| --- | --- | --- |
| #8 | Search **(((("Down Syndrome"[Mesh]) OR Down* Syndrome)) AND (("Alzheimer Disease"[Mesh]) OR (Alzheimer* Disease OR dementia))) AND ((plasma tau OR serum tau OR blood tau or plasma total tau OR plasma phosphorylated tau OR P-T181 OR P-S-396))** Sort by: **PublicationDate** | [29](https://www.ncbi.nlm.nih.gov/pubmed/?cmd=HistorySearch&querykey=26) |
| #7 | Search **(plasma tau OR serum tau OR blood tau or plasma total tau OR plasma phosphorylated tau OR P-T181 OR P-S-396)** Sort by: **PublicationDate** | [6395](https://www.ncbi.nlm.nih.gov/pubmed/?cmd=HistorySearch&querykey=25) |
| #6 | Search **("Alzheimer Disease"[Mesh]) OR (Alzheimer* Disease OR dementia)** Sort by: **PublicationDate** | [227405](https://www.ncbi.nlm.nih.gov/pubmed/?cmd=HistorySearch&querykey=22) |
| #5 | Search **Alzheimer* Disease OR dementia** Sort by: **PublicationDate** | [227405](https://www.ncbi.nlm.nih.gov/pubmed/?cmd=HistorySearch&querykey=21) |
| #4 | Search **"Alzheimer Disease"[Mesh]** Sort by: **PublicationDate** | [84328](https://www.ncbi.nlm.nih.gov/pubmed/?cmd=HistorySearch&querykey=20) |
| #3 | Search **("Down Syndrome"[Mesh]) OR Down* Syndrome** Sort by: **PublicationDate** | [44070](https://www.ncbi.nlm.nih.gov/pubmed/?cmd=HistorySearch&querykey=19) |
| #2 | Search **Down* Syndrome** Sort by: **PublicationDate** | [44070](https://www.ncbi.nlm.nih.gov/pubmed/?cmd=HistorySearch&querykey=18) |
| #1 | Search **"Down Syndrome"[Mesh]** Sort by: **PublicationDate** | [23226](https://www.ncbi.nlm.nih.gov/pubmed/?cmd=HistorySearch&querykey=17) |

**Supplemental Table 2.** Search strategy for tau studies in Pubmed
