# Supplementary material for: Plasma amyloid and tau as dementia biomarkers in Down syndrome: Systematic review and meta‐analyses
Source: Dev Neurobiol. 2019 Sep 11;79(7):684–98. doi: 10.1002/dneu.22715 (PMC6790908; doi:10.1002/dneu.22715)
Supplement: Supplementary file 7 [file DNEU-79-684-s007.docx]

**Supplemental Figure 1.** Funnel plot of studies comparing Aβ40 in DS individuals and NC.

**Abbreviations:** DS = Down syndrome; NC = Normal controls

**Supplemental Figure 2.** Funnel plot of studies comparing Aβ42 in DS individuals and NC.

**Abbreviations:** DS = Down syndrome; NC = Normal controls

**Supplemental Figure 3.** Funnel plot of studies comparing Aβ40 in demented and non-demented DS individuals.

**Abbreviations:** DS = Down Syndrome

**Supplemental Figure 4.** Funnel plot of studies comparing Aβ42 in demented and non-demented DS individuals.

**Abbreviations:** DS = Down syndrome

**Supplemental Figure 5.** Funnel plot of studies comparing Aβ ratios in demented and non-demented DS individuals.

**Abbreviations:** DS = Down syndrome
